# Career Calling as the Mediator and Moderator of Job Demands and Job Resources for Job Satisfaction in Health Workers: A Cross-Sectional Study

**DOI:** 10.3389/fpsyg.2022.856997

**Published:** 2022-05-10

**Authors:** Xianhong Huang, Hanlin Chen, Yuan Gao, Jin Wu, Ziling Ni, Xiaohe Wang, Tao Sun

**Affiliations:** ^1^Department of Health Policy and Management, School of Public Health, Hangzhou Normal University, Hangzhou, China; ^2^Department of Quality Control and Service lmprovement, The Cancer Hospital of the University of Chinese Academy of Sciences (Zhejiang Cancer Hospital), Hangzhou, China

**Keywords:** health workers, job satisfaction, career calling, job demands-resources model, structural equation model (SEM)

## Abstract

Job satisfaction of health professionals is a key determinant of the quality of health services and even affects the development of the healthcare system. In this study, we sought to explore the mechanism by which job demands, job resources, and career calling affect the job satisfaction of health professionals. Our findings may provide insights for increasing their job satisfaction and improving the quality of health services. We conducted a questionnaire survey of 1,117 health workers in Hangzhou; *t*-test, Chi-squared analysis, hierarchical linear regression was used to analyze the state of job satisfaction of health personnel and the associated factors; path analysis with the Structural Equation Model was used to explore and verify the effects of job resources, demands, and career calling on job satisfaction, as well as their mechanism. Social support, performance feedback, working conditions, and career calling had significant positive effects on job satisfaction of health professionals, whereas work-family conflict and emotional requirements for work had significant negative effects. Path analysis indicated that job resources, demands, and career calling directly affected job satisfaction; job resources and demands showed indirect effects on job satisfaction with career calling as a mediator. Career calling had a positive moderating effect in the path of “job resources–job satisfaction,” and a negative moderating effect in the path of “job demands–job satisfaction.” In conclusion, hospital administrators should provide more job resources for health workers and formulate reasonable job demands while paying close attention to work-related pressure. Hospital administrators and health departments need to improve hospital policies and inculcate a sense of belonging and career calling among health professionals. Education and evaluation of career calling need to be accorded more attention so that healthcare workers can perceive a stronger sense of calling and achievement, and hence a higher degree of job satisfaction.

## Introduction

Job satisfaction of health workers refers to their inner feeling and attitude toward their job and other work-related elements, such as the work environment, which reflects their subjective sense of satisfaction with the job (Ye and Feng, 2009). In China, it has been demonstrated that job satisfaction among health professionals was not high. For example, it was reported that only 25% of nurses working at a hospital feel satisfied with their current job (Chen et al., 2021). Another study reported a medium level of work-burnout and a high tendency for quitting among nurses working in an oncology department (Jiang et al., 2019). In addition, a cross-sectional study of essential public health practitioners in primary care revealed only a moderate level of job satisfaction among health professionals (Chen et al., 2021). Health professionals with a high level of job satisfaction are more likely to provide high-quality healthcare for patients. On the other hand, a low level of job satisfaction may reflect decision-making and hospital management problems, adversely affecting the quality and efficiency of health services, harming doctor–patient relationships, and reducing patient satisfaction (Zhang et al., 2021). Therefore, investigating the factors that influence the job satisfaction of health professionals can help inform healthcare policies and provide meaningful insights for increasing their job satisfaction.

The Job Demands-Resources (JD-R) model is a popular conceptual framework used in professional burnout (Demerouti et al., 2001). The common factors related to job satisfaction were work pressure, interpersonal relations, rewards from work, working conditions, and social support (Wang et al., 2017a). However, according to the JD-R model, job satisfaction of employees is related to the demands of the organization and the resources it provides (Potocka and Waszkowska, 2013). Job demands can create pressure and a feeling of burnout, intensifying conflicts, and lowering job satisfaction (Drüge et al., 2021), whereas abundant resources can stimulate the dedication and engagement of employees, improving their job satisfaction (Flores et al., 2021). Along with the socio-economic development, individuals choosing a career now focus more on job satisfaction brought about by motivation, work meaning, and social responsibility, making the notion of career calling receive wide attention. Career calling refer to a solid inner passion and drive toward a particular field of work (Dobrow and Tosti-Kharas, 2011). Studies have demonstrated a close association between the sense of career calling and job satisfaction (Duffy et al., 2015). In addition, the sense of career calling has been shown to drive autonomic motivation and hence increase an individual’s job satisfaction (Shi et al., 2018).

Several studies have explored the determinants of job satisfaction of health personnel or investigated the relationship between job demands, resources, and job satisfaction (Yeh, 2015; Drüge et al., 2021; Flores et al., 2021). However, these studies have merely touched upon the relationship between these three elements without exploring the mechanism of the associated pathways. In addition, few studies have investigated the relationship among the four factors simultaneously (i.e., job demands, resources, career calling, and satisfaction). Furthermore, those studies on satisfaction using the JD-R have focused on teachers or civil servants, while there is a paucity of studies in healthcare settings. Thus, based on the JD-R model, the present study introduced career calling as the mediating and moderating variable in the relations among job demands, resources, and satisfaction of health personnel. The objective was to analyze the mediating/moderator role of career calling in the relationship between the JD-R model and job satisfaction. The purpose of the present study was to help understand and clarify the impact of job resources, job demands, and calling on the job satisfaction of medical staff and the underlying mechanism; in addition, we sought to explore the dual effects of occupational calling.

The rest of this paper is organized as follows: First, we review the JD-R model and the theory and research on the relationship between career calling and satisfaction to provide the basis for this study. Then, we propose the research model and research hypotheses. Next, we describe the research methods, data collection, and measurement. In the next section, we present the results. Finally, we discuss the findings, theoretical and practical implications, limitations of the study, and opportunities for future research.

## Theory and Hypotheses

### Effects of Job Demands and Resources on Job Satisfaction

With strides made in positive psychology, some researchers have unraveled certain patterns while investigating the relationship between different variables and positive outcomes using the JD-R model. The job demands are the standards that employees must meet to be competent at work, which is the source of employee work pressure. It specifically refers to the psychological, physical, social, and organizational requirements of the work itself, which require employees to respond continuously in terms of psychology, physical strength, and skills (Bakker et al., 2004; Yuan, 2018; Xian, 2019). According to the JD-R model, high job demands can exhaust the mental and physical resources of employees, causing negative outcomes, such as low job satisfaction and high separation rate. For instance, it was reported that balancing the job demands, job control, and social support can enhance job satisfaction of nurses (Bagheri Hossein Abadi et al., 2020). Besides, a cross-sectional study of workers in enterprises found that the job demands were negatively associated with their health, while job resources were positively related to their health and well-being (Lopez-Martin and Topa, 2019).

The job resources are the support employees get at work and the sources of employee motivation, which specifically refer to the psychological, physical, social, and organizational resources that employees can get from the work level. Job resources include remuneration, career development opportunities, job security, social support, work autonomy, and performance feedback. These resources can help employees devote themselves to work, achieve work goals, reduce work pressure, and promote personal career growth and development (Tims et al., 2012; Wang et al., 2012). Besides, job resources were identified as the moderator in the relationship between job demands and occupational strain, whereas there was no significant association between occupational strain and job satisfaction (Ghanayem et al., 2020). Another study demonstrated a correlation of job resources and job demands with burnout, turnover intention, and job satisfaction (Scanlan and Still, 2019). In addition, the job demands were found to directly affect the degree of job burnout, and job resources showed a moderating effect in the relationship between job demands and burnout (Xian and Zhai, 2019).

The above findings indicate an association of job demands and resources with the job satisfaction of health personnel—the job satisfaction increases as job resources grow. At the same time, it decreases as job demands rise. Thus, we predict the following: Hypothesis 1 (H_1_): Job resources positively affect the job satisfaction of health workers. Hypothesis 2 (H_2_): Job demands negatively affect the job satisfaction of health workers.

### The Influence of Career Calling on Job Satisfaction and Its Mediating and Moderating Effects

Career calling is a passion or driving force that motivates individuals to act with a clear vision and a sense of mission (Ye and Feng, 2009). Career calling has effects on job satisfaction. For instance, a qualitative study demonstrated that people who have a strong sense of mission can have high job satisfaction even when facing discrimination against their careers (Greene and Robbins, 2015). In another study, the group with a strong sense of career calling showed greater job satisfaction compared to the group with a weak sense of career calling (Mao et al., 2019). Some researchers have pointed out that job satisfaction is a complex variable which is influenced by both work environment-related factors and individual personality traits (Fors Brandebo et al., 2019). Another study by Hirschfeld (2000) further pointed out that job satisfaction can be divided into two dimensions: extrinsic and intrinsic. Based on self-determination theory (Deci and Ryan, 2012) and some literature review (Duffy and Sedlacek, 2010; Duffy et al., 2012a), career calling was found to be associated with career development and job satisfaction. For example, studies have found that career calling can improve individual job commitment and satisfaction and reduce individual turnover intention and absenteeism (Wrzesniewski et al., 1997; Duffy et al., 2012b). Besides, Peterson et al. found that career calling can lead to high levels of job satisfaction. Another explanation for the close relationship between career calling and job satisfaction is the theory of prosocial work motivation, which defines it as “a momentary egocentric state in which employees are focused on making positive contributions to the lives of others” (Grant and Berry, 2011). In previous studies, higher levels of prosocial work motivation were associated with higher levels of job satisfaction, job performance, and task efficiency (Grant et al., 2008; Grant and Berry, 2011). Based on the above evidence, we predict the H_3_: Career calling has positive effects on job satisfaction of health workers.

Besides, career calling of health workers can play a role in the relationship between job resources and job satisfaction. In a study of primary and secondary school teachers, their professional mission was found to partially mediate the relationship between character strengths and work engagement (Hu et al., 2021). Another study of rural teachers showed that the sense of professional mission mediates the relationship between perceived social support and turnover intention (Li, 2021). In a study of frontline nurses deployed in designated hospitals for COVID-19 pneumonia, the professional mission of the nurses was found to partially mediate the perceived stress and psychological resilience (Xu et al., 2020). A recent study reported that COVID-19 pandemic criticality positively affected the work fatigue and career calling, and that the career calling mediated the direct link between COVID-19 pandemic criticality and work fatigue (Zhou, 2022). Therefore, we predict H_4a_: Career calling of health workers is a mediator in the relationship between job resources and job satisfaction and H_4b_: Career calling of health workers is a mediator in the relationship between job demands and job satisfaction.

Furthermore, career calling has been found to positively moderate the relationship between professional resources and job satisfaction. A study found a positive moderating effect of career calling on the relationship between job requirements and job burnout (Xie, 2017). Another study found that the career calling of teachers had a positive moderating effect on the relationship between occupational stress and job burnout (Zhang et al., 2020). According to another study, the sense of calling moderates the indirect effect of job autonomy and career commitment through job crafting. The indirect effect is more potent in people with a higher sense of calling than those with a lower sense of calling (Chang et al., 2021). We, therefore, predict H_5a_: Career calling of health workers is a positive moderator in the relationship between job resources and job satisfaction and H_5b_: Career calling of health workers is a negative moderator in the relationship between job demands and job satisfaction.

**Figure 1:**
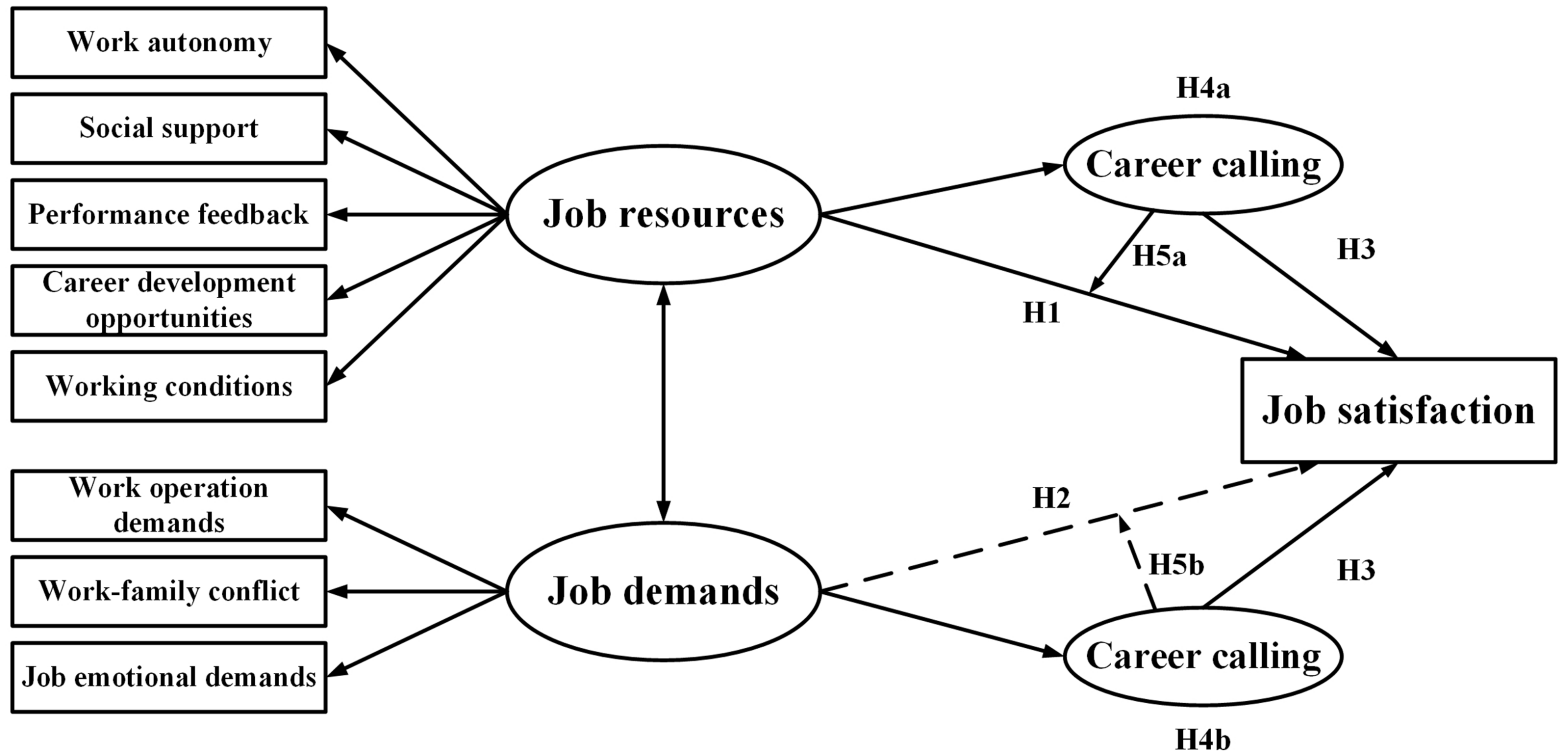
Model for the mechanism of the influence of job demands-resources on job satisfaction.

To sum up, there are correlations among job demands, job resources, career calling, and satisfaction. Besides, career calling is the mediator and/or moderator in the relationship between certain antecedent variables (e.g., job resources, work-life conflicts, etc.) and certain outcomes (e.g., job satisfaction, performance, work-burnout, etc.). Additionally, several studies have indicated that a single variable could act both as a mediator and a moderator (MacKinnon, 2008; Dicke et al., 2014; Attar-Schwartz, 2015; Liu and Li, 2017; Liu, 2019; Peng et al., 2021). The level of career calling is affected by factors, such as circumstances and self-efficacy, and at the same time it mediates/moderates the relationship between circumstances and job satisfaction. By and large, career calling can influence job satisfaction *via* various pathways, and there is no conflict between the two paths above. Therefore, they both were examined in this study. The theoretical model is shown in Figure 1.

## Materials and Methods

### Participants and Procedures

Hangzhou is an economically developed municipality located on the southeastern coast of China, with a *per capita* GDP of US$9,696 in 2020. There are more than 20 tertiary public hospitals, 30 secondary public hospitals, and more than 60 community health centers in Hangzhou.

In this study, we conducted a survey of health professionals working in health facilities of different levels in Hangzhou using stratified random sampling methodology. First, stratified sampling was conducted according to the levels of health facilities. Four tertiary hospitals, seven secondary hospitals, and 15 community health centers from different counties were selected by random sampling. Then health workers in the selected health facilities were selected using random sampling methodology. Thus, approximately 105 workers were selected from each tertiary hospital, approximately 55 from each secondary hospital, and approximately 25 from each community health center. The inclusion criteria for study participants were health workers directly involved in providing medical and/or nursing services to patients, including medical doctors, nurses, and medical technicians; those working on the day of the study and had worked for more than half a year in the facility; and consenting to participate in the study. The exclusion criteria were health workers from other facilities, interns, or trainees; health workers who were off work or did not consent to participate; administrative or logistic personnel not majoring in medicine.

Prior to the full-scale survey, a preliminary survey was conducted to establish the specific implementation of the research and the final version of the questionnaire. The formal investigation was conducted by two fellows and five graduates with sufficient field investigation experience from 1 July to 30 September 2020. In addition, the investigators were trained to use the same standards and methods; during the on-site investigation process, a one-to-one method was used. Informed consent of the participants was obtained before the investigation, and necessary explanations were provided during the investigation. After the completion of the survey, the questionnaire was checked to determine whether it met the requirements. After completing the survey, the questionnaires were uniformly numbered, and double entered to ensure the accuracy of data entry. A total of 1,300 questionnaires were distributed on-site, and 1,223 questionnaires were completed, with a recovery rate of 94.1%; 1,117 qualified questionnaires were recovered, with an effective rate of 91.3%. The characteristics of the final 1,117 participants are listed in Table 1.

**Table 1:** Descriptive statistics of the sample (*N* = 1,117).

Characteristic	Category	Frequency (*N*)	Composition ratio (%)	Characteristic	Category	Frequency (*N*)	Composition ratio (%)
Gender	Male	303	27.1	Work experience (years)	<5 years	346	31.0
	Female	814	72.9		6–10	309	27.7
Marital status	Unmarried	290	26.0		11–15	207	18.5
	Married	809	72.4		16–20	117	10.5
	Divorced/Other	18	1.6		≥21	138	12.4
Age (years)	<29	394	35.3	Post	Clinical doctor	373	33.4
	30–39	470	42.1		Nurse	530	47.4
	40–49	211	18.9		Medical technician	214	19.2
	≥50	42	3.8	Position	No position	862	77.2
Academic degree	College and below	197	17.6		Medical group leader	121	10.8
	Undergraduate	732	65.5		Head of department or higher	134	11.5
	Masters or above	188	16.8	Department	Internal medicine	253	22.6
Professional title	To be assessed	139	12.4		Surgery	153	13.7
	Primary title	434	38.9		Emergency	59	5.3
	Middle title	384	34.4		Pediatrics	109	9.8
	Vice-senior title	132	11.8		Gynecology and obstetrics	90	8.1
	Senior title	28	2.5		Intensive care unit	12	1.1
Availability of budgeted posts	Yes	691	61.9		Medical technology	151	13.5
	No	424	38.0		Other	290	26.0
Hospital level	Grade III	718	64.3				
	Grade II	236	21.1				
	Community health service center	163	14.6				

### Measures

Internal consistency (Cronbach’s *α* coefficient) and composite reliability (CR) were used to evaluate the reliability of the questionnaire, and the validity of the measurement was evaluated using content and aggregation validity. The questionnaire included the following sections: Sociodemographic information questions, Job demand measure, Job resource measure, Career calling measure, and a measure of job satisfaction.

#### Sociodemographic Information Form

The general survey form comprised of 11 items, including: sex (male, female), age (≤29, 30–39, 40–49, and ≥50), marital status (single, married, and divorced/other), level of education (junior college or lower, undergraduate degree, and graduate degree or higher), position (no position, medical group leader, and head of department or higher), title (no title, junior, middle, sub-senior, and senior), post (clinical doctor, nurse, and medical technician), type of employment (budgeted post, contract job), department, years of experience (≤5, 6–10, 11–15, 16–20, and ≥21), and level of hospital (tertiary hospital, secondary hospital, and community health center).

#### Job Demand Scale

The Job demand scale (JDS) amended by Li Meng was used (Li, 2006). It comprised of three sub-scales: demands on work operations, demands on feelings, and work–family conflict, which include 11 items. The items were scored using a 5-point Likert scale (from 1 = Strongly Disagree to 5 = Strongly Agree). The Cronbach’s alpha for JDS was 0.888, and the Cronbach’s alpha for the dimensions were between 0.847 and 0.930. The CR values of JDS were 0.751, factor loading was from 0.607 to 0.920, the AVE value was 0.513, the correlation coefficient between each item and the overall score was 0.472–0.777 (*p* < 0.001), indicating good reliability and validity of the measures (Table 2).

**Table 2:** Measurement items and results of reliability and validity analysis of the questionnaire (*N* = 1,117).

Construct	Dimension	Measurement items	Cronbach’s α	Load^a^	Correlation coefficient	AVE	CR	Overall α value
Job resources	Work autonomy	B1 I determine the progress of work on my own	0.913	0.840	0.695^**^	0.712	0.925	0.964
		B2 I am entitled to decide how to carry out my work		0.891	0.727^**^			
		B3 I can organize work-related affairs by myself		0.863	0.729^**^			
	Social support	B4 I can seek help from leaders when encountering difficulties at work	0.915	0.854	0.802^**^			
		B5 My leaders care about my physical and mental health		0.891	0.843^**^			
		B6 My leaders can improve my relationships with colleagues		0.914	0.855^**^			
		B7 I can get help from colleagues		0.867	0.726^**^			
		B8 My colleagues are friendly to me		0.825	0.699^**^			
	Performance feedback	B9 I know precisely comments of leaders on my performance	0.945	0.766	0.842^**^			
		B10 My leaders often offer feedbacks beneficial to career development		0.757	0.871^**^			
		B11 Performance feedbacks from my leaders are well explained		0.711	0.875^**^			
	Career development opportunities	B12 My job provides me with opportunities to learn professional skills including new clinical techniques and new projects	0.910	0.545	0.826^**^			
		B13 The hospital I work at organizes various activities including lectures and training		0.846	0.769^**^			
		B14 The hospital I work at organizes internal recruitment or selection for certain posts and titles		0.833	0.787^**^			
		B15 The hospital I work at encourages us to apply for scientific projects		0.782	0.733^**^			
	Working conditions	B16 In the department I work at, the technologies and equipment can meet the needs of patients	0.883	0.591	0.765^**^			
		B17 The department I work at is staffed reasonably		0.789	0.745^**^			
		B18 The department I work at has a good cultural atmosphere		0.701	0.810^**^			
		B19 The hospital I work at can deal with violence happening in the workplace timely		0.699	0.775^**^			
Job demands	Work operation requirements	C1 My job requires me to work in a fast-paced environment	0.876	0.768	0.623^**^	0.513	0.751	0.888
		C2 My job requires me to be very hard-working		0.865	0.612^**^			
		C3 I have a heavy workload		0.720	0.735^**^			
		C4 My job has strict requirements for the quality of work		0.852	0.637^**^			
		C5 My job requires me to pay attention to the safety in operation		0.732	0.472^**^			
	Work-family conflict	C6 Requirements of my job affected my family life	0.930	0.891	0.732^**^			
		C7 Time spent in my job made me fail to undertake family responsibilities		0.920	0.756^**^			
		C8 I failed to do what I wanted to do at home due to requirements of my job		0.903	0.739^**^			
	Emotional requirements for work	C9 My job requires me to deal with various demands of patients or their relatives	0.847	0.862	0.740^**^			
		C10 My work involves getting along with patients with poor compliance		0.870	0.721^**^			
		C11 My work put me under heavy pressure		0.607	0.777^**^			
Career calling	Career calling	E1 I perceive a calling as a health worker	0.913	0.861	0.821^**^	0.615	0.862	0.913
		E2 I have found the career in which I have a calling		0.859	0.839^**^			
		E3 I am trying to fathom the mission of health workers		0.854	0.874^**^			
		E4 I am seeking my mission as a health worker		0.839	0.876^**^			

CR, composite reliability; AVE, average variance extraction.

a All load values are significant at the 0.001 level.

** *p* < 0.01, two-tailed test.

#### Job Resource Scale

We added the dimension of working conditions on the basis of the Job Resource Scale (JRS) established by Li Jienan (Li, 2011). Therefore, the scale consisted of five dimensions (i.e., work autonomy, social support, performance feedback, career development, and working conditions) with 19 items. A 7-point Likert scale was used (from 1 = Strongly Disagree to 7 = Strongly Agree) with higher scores representing more job resources. In this study, the Cronbach’s alpha for JRS was 0.964, and the Cronbach’s alpha for the dimensions were between 0.883 and 0.945. The CR values of JRS were 0.925, factor loading was from 0.545 to 0.914, the AVE value was 0.712, the correlation coefficient between each item and the overall score was 0.695–0.875 (*p* < 0.001), indicating good reliability and validity of the measures (Table 2).

#### Career Calling Scale

The CQ12 scale measure of career calling was used (Dobrow and Tosti-Kharas, 2011). It contained only one dimension and four items each of which was scored on a 5-point Likert scale (from 1 = Strongly Disagree to 5 = Strongly Agree). The Cronbach’s alpha was 0.913. The CR values of Career Calling Scale (CCS) were 0.862, factor loading was from 0.839 to 0.861, the AVE value was 0.615, the correlation coefficient between each item and the overall score was 0.821–0.876 (*p* < 0.001), indicating good reliability and validity of the measures (Table 2).

#### Job Satisfaction Scale

The Job satisfaction scale had only one item, i.e., “I am very satisfied with my current job” scored on a 5-point Likert scale (from 1 = Strongly Disagree to 5 = Strongly Agree). Many scholars believe that, compared to scales with multiple dimensions and items, single-item scales of overall job satisfaction have a greater validity (Scarpello and Campbell, 1983; Wanous et al., 1997; Su and Bozeman, 2009).

### Statistical Analysis

Statistical analyses were performed using SPSS 26.0. Data were analyzed using descriptive statistics such as frequency and constituent ratio. Differences in scores on job demands-resources, career calling, and job satisfaction of health personnel with different characteristics were analyzed using *t*-test and one-way ANOVA. Hierarchical multiple regression analysis (enter method) was used to analyze the main factors influencing job satisfaction of health professionals. A forward stepwise multiple regression analysis using job satisfaction as a dependent variable was applied. All variables that may potentially influence job satisfaction were added in the model to deal with any possible confounding.

The Structural Equation Model (SEM) was constructed using AMOS 22.0 to explore the specific mechanism of the (direct or indirect) influence of job demands, resources, and calling on job satisfaction, as well to calculate the corresponding effect sizes. The Bootstrap method was used to verify the mediating effect of career calling; the two-step technique proposed by Ping (1995) was used to analyze its moderating effect. The two paths involved in the model were: job resources → career calling → job satisfaction, and job demands → career calling → job satisfaction; we judged the fit of the hypothesized model, and modified it with modification indices, so as to make the modified model fit the best with the sample data.

## Results

### Descriptive Statistics and Correlation Analysis of Health Workers

The dimensions of job resources and career calling showed a positive correlation with job satisfaction, and the correlation coefficients between dimensions were between *r* = 0.322–0.498 (all *p* < 0.01). Among the dimensions of job requirements, job operational requirements showed positive correlation with job satisfaction, with a correlation coefficient of *r* = 0.094 (*p* < 0.01); work–family conflict, job emotional demands showed negative correlation with job satisfaction, *r* = −0.157 to −0.217 (*p* < 0.01), indicating suitability for further regression and structural equation model analysis (Table 3).

**Table 3:** Mean, standard deviation, and correlation coefficient of each variable.

Variable	1	2	3	4	5	6	7	8	9	10
1. Work autonomy	/									
2. Social support	0.646^**^	/								
3. Performance feedback	0.650^**^	0.805^**^	/							
4. Career development opportunities	0.565^**^	0.719^**^	0.758^**^	/						
5. Working conditions	0.584^**^	0.766^**^	0.774	0.783^**^	/					
6. Work operation requirements	0.300^**^	0.428^**^	0.319^**^	0.438^**^	0.352^**^	/				
7. Work–family conflict	0.028	−0.084^**^	−0.037	0.004	−0.085^**^	0.295^**^	/			
8. Emotional requirements for work	0.117^**^	0.097^**^	0.061^*^	0.146^**^	0.061^*^	0.514^**^	0.578^**^	/		
9. Career calling	0.348^**^	0.422^**^	0.428^**^	0.410^**^	0.450^**^	0.333^**^	0.067^*^	0.126^**^	/	
10. Job satisfaction	0.273^**^	0.424^**^	0.433^**^	0.366^**^	0.461^**^	0.050	−0.312^**^	−0.242^**^	0.317^**^	/
Mean ± Standard deviation	5.11 ± 1.38	5.67 ± 1.09	5.23 ± 1.38	5.52 ± 1.20	5.44 ± 1.16	4.29 ± 0.58	3.40 ± 1.00	3.98 ± 0.82	3.99 ± 0.70	3.42 ± 0.88

^**^p < 0.01, two-tailed test.

### Comparison of Job Satisfaction of Health Workers With Different Demographic Characteristics

Results demonstrated significant differences (*p* < 0.05) in the scores of job satisfaction of health professionals with different level of marital status, education, post, and position (Table 4).

**Table 4:** One-way ANOVA of job satisfaction of health workers with different demographic characteristics.

Variable	*n*	Job satisfaction	*F*-value	*p-*Value
*Marital status*				
Unmarried	290	3.25 ± 0.878	4.185	0.015
Married	809	3.41 ± 0.814		
Divorced/Other	18	3.39 ± 0.832		
*Level of education*				
Junior college or lower	197	3.59 ± 0.885	5.087	0.006
Undergraduate degree	732	3.37 ± 0.888		
Graduate degree (master’s degree or doctoral degree)	180	3.41 ± 0.870		
*Post*				
Clinical doctor	373	3.36 ± 0.891	3.407	0.033
Nurse	530	3.40 ± 0.888		
Medical technician	214	3.55 ± 0.869		
*Position*				
No position	862	3.38 ± 0.880	8.811	<0.001
Medical group leader	121	3.37 ± 0.886		
Head of department or higher	134	3.78 ± 0.811		

### Hierarchical Multiple Regression Analysis of Job Satisfaction

First, dummy variables were used to represent unordered categorical data that were statistically significant in the one-way ANOVA of job satisfaction, including marital status, level of education, post, and position. Then hierarchical multiple regression analysis was conducted using job satisfaction as the dependent variable, with the four blocks being: (1) demographic characteristics; (2) demographic characteristics + job resources; (3) demographic characteristics + job resources + job demands; (4) demographic characteristics + job resources + job demands + career calling. Results indicated that Δ*R*^2^ were statistically significant when demographic characteristics, job resources, job demands, and career calling were entered into the regression equation. Specifically, after comparing the changes in the Δ*R^2^*, job resources showed a stronger effect on job satisfaction than demographic characteristics, job demands, and career calling, accounting for 26.5% of the variance in job satisfaction (Table 5).

**Table 5:** Hierarchical multiple regression analysis of job satisfaction.

Variables	First block	Second block	Third block	Fourth block
	Standardized *Beta*	Standardized *Beta*	Standardized *Beta*	Standardized *Beta*
*Marital status (single = reference group)*				
Married	0.072^*^	0.067^*^	0.073^**^	0.063^*^
Divorced or other	0.035	0.054^*^	0.060^*^	0.058^*^
*Level of education (junior college or lower = reference group)*				
Undergraduate degree	−0.129^**^	−0.058	−0.036	−0.027
Graduate degree (master’s degree or doctoral degree)	−0.060	−0.017	0.003	0.016
*Post (clinical doctor = reference group)*				
Nurse	0.038	−0.045	−0.046	−0.033
Medical technician	0.097^**^	0.058^*^	0.047	0.046
*Position (no position = reference group)*				
Medical group leader	−0.007	−0.028	−0.015	−0.017
Head of department or higher	0.093^**^	0.082^**^	0.081^*^	0.082^**^
*Job resources*				
Work autonomy		−0.025	−0.002	−0.009
Social support		0.117^*^	0.121^*^	0.117^*^
Performance feedback		0.182^**^	0.162^**^	0.142^**^
Career development opportunities		−0.134^**^	−0.082	−0.084
Working conditions		0.393^**^	0.358^**^	0.320^**^
*Job demands*				
Work operation requirements			−0.025	−0.050
Work–family conflict			−0.104^**^	−0.113^**^
Emotional requirements for work			−0.107^**^	−0.104^**^
*Career calling*				
Career calling				0.156^**^
*R^2^*	0.032	0.297	0.335	0.353
*F*	4.550^**^	35.885^**^	34.613^**^	35.211^**^
△*R^2^*	0.032	0.265	0.038	0.018
△*F*	4.550^**^	83.315^**^	20.751^**^	30.116^**^
VIF_max_	2.082	3.965	4.085	4.108

VIF_max_, Variance inflation factor maximum.

* *p* < 0.05;

** *p* < 0.001.

After comparing the independent variables in the fourth block, the results showed that: married health professionals scored higher with single workers as the reference group; health professionals who were head of department or higher scored higher with workers having no position as the reference group. In terms of the dimension of job resources (*β* = 0.117, *p* = 0.015), job satisfaction increased with increase in scores for social support, performance feedback, and working conditions (*β* = 0.117, *p* < 0.05; *β* = 0.142, *p* = 0.004; *β* = 0.320, *p* < 0.001); the effects of work autonomy and career development opportunities on job satisfaction were not statistically significant (*p* > 0.05), with respect to the dimension of job demands, job satisfaction decreased with increase in scores for work–family conflict and emotional requirements for work (*β* = −0.113, *p* < 0.001; *β* = −0.104, *p* = 0.002); regarding the dimension of career calling, job satisfaction increased with increase in the score for career calling (*β* = 0.156, *p* < 0.001).

### Model Construction

**Figure 2:**
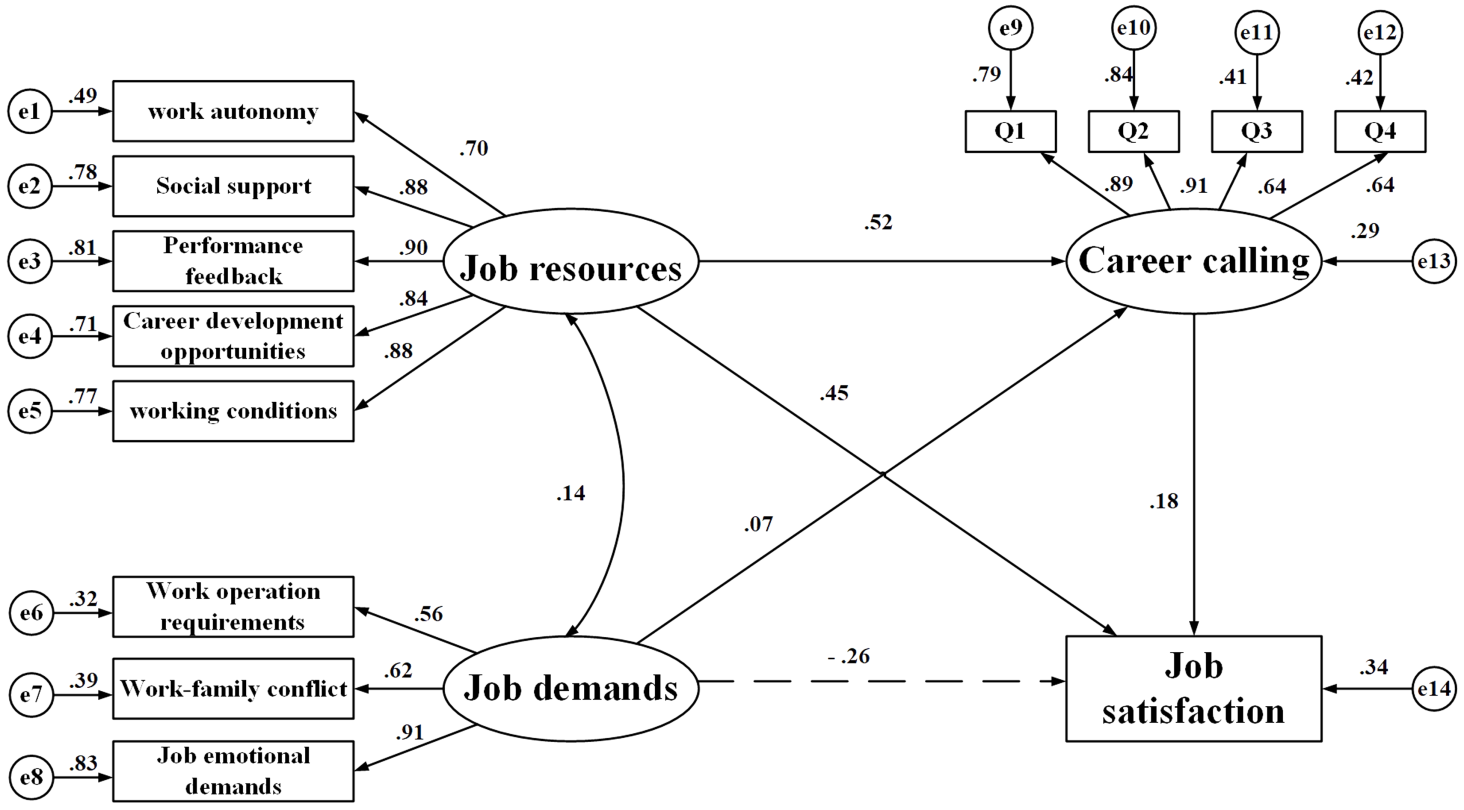
Model for the mechanism of the effects of factors on job satisfaction.

Structural Equation Model was constructed using job resources, job demands, and career calling as latent variables, while dimensions of job resources and job demands, as well as job satisfaction were used as the measured variables (Figure 2). The results of model fit indicated that, though the paths were statistically significant (for job demands → career calling, *p* = 0.014; for other paths, *p* < 0.001), the fit indices did not reach ideal values. As scholars (Bollen and Stine, 1992; Enders, 2005) have suggested, poor model fit may be attributable to a sample size that is too large or a poor model. Based on this, the model was modified using the *p* value correction method proposed by Bollen and Stine (1992). After 2,000 iterations of Bootstrap resampling, the results showed that, for the modified model, Bollen-Stine bootstrap *p* value was 0.000, *χ*^2^/*df* (1.40) was located between 1 and 3, goodness of fit (GFI), adjusted goodness of fit (AGFI), normed fit index (NFI), comparative fit index (CFI), and Tucker–Lewis index (TLI) were >0.9, and the root mean square error of approximation (RMSEA; 90% CI) was <0.08. This demonstrated that the relatively poor model fit was attributable to the large sample size, rather than the model itself. This means that the path analysis model for job satisfaction had a good overall fit (Table 6).

**Table 6:** Results for SEM fit.

Fit indices	Standards of fit indices	Original model	B-S modified model
*χ*^2^/*df*	1 < *χ*^2^/*df* < 3 good	29.100	1.403
RMSEA (90% CI)	<0.08 acceptable	0.159	0.019
GFI	>0.9 acceptable	0.840	0.991
AGFI	>0.9 acceptable	0.757	0.982
NFI	>0.9 acceptable	0.822	0.991
CFI	>0.9 acceptable	0.827	0.998
TLI	>0.9 acceptable	0.775	0.997

### Path Analysis for Job Satisfaction

By standardizing the effects, we found that job resources and career calling had positive effects on job satisfaction of health professionals, with standardized path coefficients being 0.545 and 0.184 (*p* < 0.001), respectively. Job demands showed negative effects on job satisfaction, with the coefficient being −0.242 (*p* < 0.001). Job resources and demands contributed positive effects on career calling, with the coefficients being 0.521 (*p* < 0.001) and 0.074 (*p* = 0.002), respectively. Additionally, job resources and demands had not only direct effects on job satisfaction (effect sizes: 0.449 and −0.256, respectively), but also indirect effects through career calling as the mediator (effect sizes: 0.096 and 0.014, respectively), supporting the hypotheses H_1_ and H_2_. Besides, career calling had only direct effect on job satisfaction (effect size: 0.184), supporting hypothesis H_3_ (Table 7).

**Table 7:** Path coefficients for job satisfaction and hypothesis verification.

Relations between variables	Standardized direct effect	Standardized indirect effect	Standardized total effect	*p*-Value	Supported hypotheses
Job resources → Job satisfaction	0.449	0.096	0.545	<0.001	H_1_
Job demands → Job satisfaction	−0.256	0.014	−0.242	<0.001	H_2_
Career calling → Job satisfaction	0.184	/	0.184	<0.001	H_3_

### Bootstrap Test for the Mediating Effect of Career Calling in the Relationships Between Job Resources-Demands and Job Satisfaction

The effect sizes of the overall effects of job resources and demands on job satisfaction were 0.545 and −0.242, respectively, while the confidence intervals did not include 0, which demonstrated the existence of overall mediating effect, which allows for further analysis. The effect sizes of the direct effects of job resources and demands on job satisfaction were 0.449 and −0.256, respectively, while the confidence intervals did not include 0. The effect sizes of the indirect effects of job resources and demands on job satisfaction were 0.096 and −0.014, respectively, while the confidence intervals did not include 0. Thus, job resources and demands showed significant direct and indirect effects on job satisfaction, both being partially mediated effects, which supported the hypothesis H_4_ (Table 8).

**Table 8:** Examining the mediating effect with Bootstrap method (standardized coefficients).

Paths	Effect of type	S. E	Effect sizes	Bias-corrected 95% CI			Percentile 95% CI		
				Lower	Upper	*p*	Lower	Upper	*p*
Job resources → Job satisfaction	Total effects	0.028	0.545	0.485	0.598	0.001	0.490	0.602	0.001
	Direct effects	0.033	0.449	0.379	0.512	0.002	0.385	0.518	0.001
	Indirect effects	0.020	0.096	0.058	0.134	0.001	0.057	0.133	0.001
Job demands → Job satisfaction	Total effects	0.040	−0.242	−0.316	−0.165	0.001	−0.319	−0.166	0.001
	Direct effects	0.040	−0.256	−0.331	−0.177	0.001	−0.332	−0.178	0.001
	Indirect Effects	0.007	0.014	0.001	0.031	0.043	0.000	0.030	0.053

### Test for Moderating Effects of Career Calling in the Relations Between Job Resources, Demands, and Job Satisfaction

**Figure 3:**
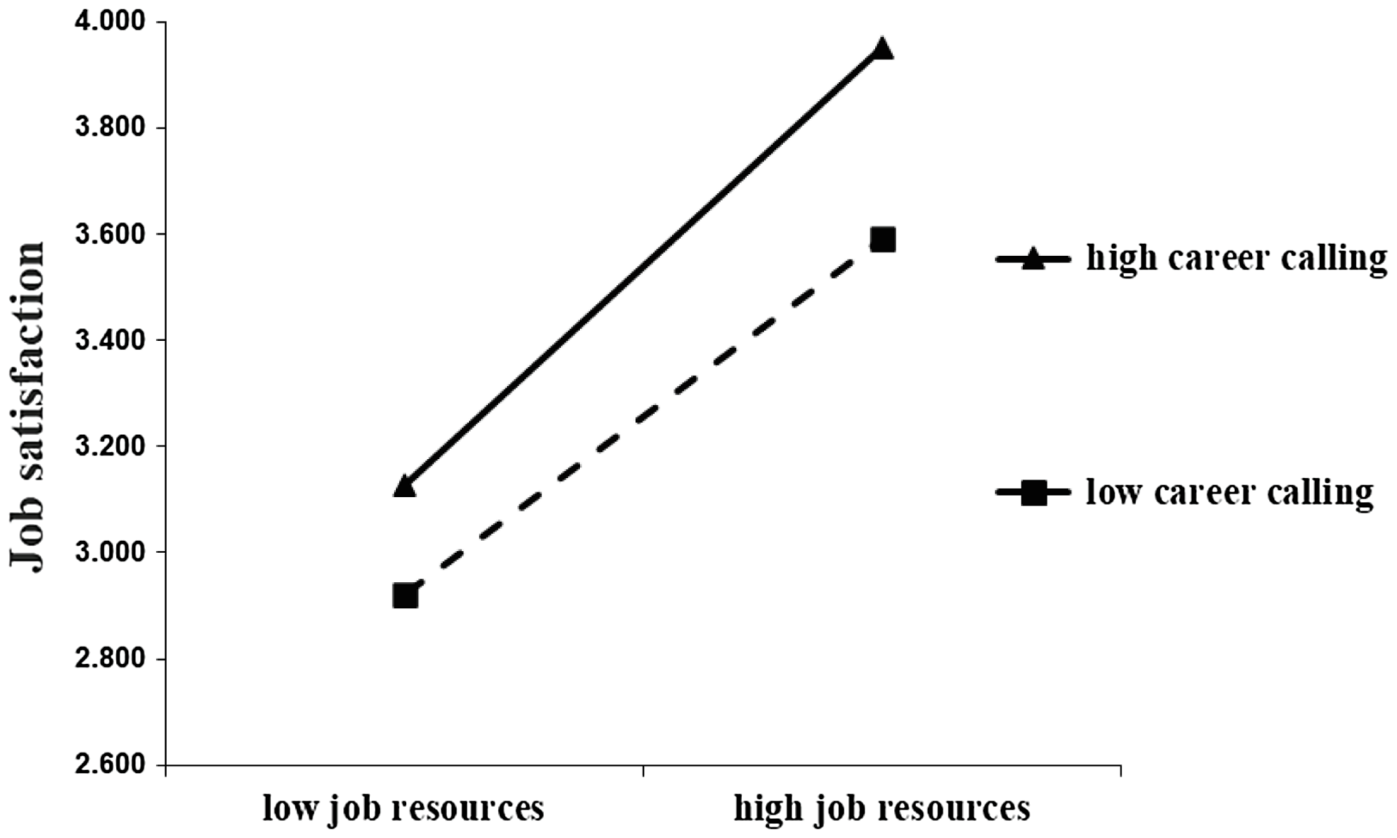
Moderating effect of career calling in the relation between job resources and job satisfaction.

**Figure 4:**
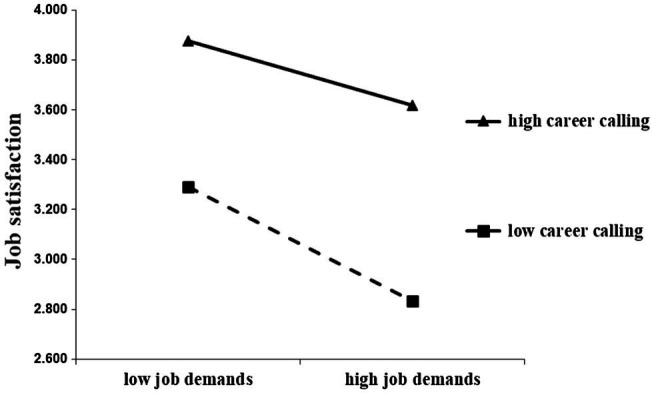
Moderating effect of career calling in the relation between job demands and job satisfaction.

The results indicated a good fit of the modified model with the data. Career calling showed a positive moderating effect in the path of “job resources–job satisfaction” (*β* = 0.527, *p* < 0.001), and a negative moderating effect in the path of “job demands–job satisfaction” (*β* = −0.209, *p* = 0.010), supporting hypothesis H_5_ (Table 9). Additionally, to further illustrate the moderating effect of career calling, the PROCESS tool was used to perform simple slope test and further examine the interaction model, and simple effect graphs were drawn using values of mean ± single standard deviation of independent variables and moderating variables in the regression equation (Figures 3, 4). As shown by the graphs, job satisfaction increased with increase in job resources, and decreased with increase in job demands, irrespective of strong or weak career calling. However, when the level of career calling was high, the positive effect of job resources on job satisfaction was more significant, and when the level of career calling was low, the negative effect of job demands on job satisfaction was more significant.

**Table 9:** Examining moderating effect.

Relations between variables	Unstd.	Std.	Standard errors	*p*	Supported hypothesis
Job resources * Career calling → Job satisfaction	0.043	0.527	0.008	<0.001	H_5_
Job demands * Career calling → Job satisfaction	−0.016	−0.209	0.006	0.010	H_5_

Unstd., Unstandardized parameter estimates; Std., Standardized parameter estimates.

## Discussion

The present study proposed a dual-action model based on the JD-R model to examine the mediating and moderating roles of career calling between job resources & job demands and job satisfaction. The results showed a positive effect of job resources and career calling on job satisfaction, while job demands were found to have a negative impact on job satisfaction. In addition, the mediating role of career calling suggested that the enrichment of job resources increased career calling, thereby promoting the job satisfaction of medical staff. At the same time, the relationship between job demands and job dissatisfaction can be mediated and weakened by the sense of career calling. When career calling is high, job resources significantly impact job satisfaction of medical staff in terms of moderating effect. Besides, job requirements have a more potent inhibitory effect on job satisfaction of medical staff when career calling is low.

### Job Resources, Job Demands, and Job Satisfaction

Several job-stress models have been developed in the field of stress management, the most representative of which are the Job Demand-Control Model (JDC; Karasek, 1979), the Job Demand-Control-Support Model (JDCS; Johnson and Hall, 1988), and the Job Demands-Resources Model (JD-R) on which this study was based (Demerouti et al., 2001). The common point of those models is that certain job characteristics (e.g., job demands) in the work settings can lead to negative stress responses. In contrast, other job characteristics (e.g., work resources, including job control, autonomy, social support, feedback, and career opportunities) can adjust or buffer the negative effects of job demands on the physical and mental health of employees (Bakker and Demerouti, 2007; Crawford et al., 2010). Our findings based on the survey of medical staff confirmed this model, i.e., the job resources positively affected job satisfaction (H_1_), and the job demands negatively affected the job satisfaction (H_2_).

Furthermore, we also found that job resources had a greater impact on the job satisfaction of health care workers than demographic characteristics, job demands, and career aspirations. This finding is consistent with the JD-R model, which proposes that valuable environment, organizational, and personal work resources can promote the completion of work goals, reduce the physical and psychological costs of employees, and stimulate their growth and development (Demerouti et al., 2001). Specifically, among the five dimensions of work resources in this study, work conditions and performance feedback showed a more significant impact on job satisfaction. Health workers with good working conditions tend to have greater job satisfaction (Mache and Vitzthum, 2012; Liang, 2020). Studies have shown that the satisfaction and motivation of employees is likely to increase when they accept, understand, and act on performance feedback (Gomez-Mejia, 1990; Singh, 1998). Besides, receiving objective, valid, and timely performance feedback can help employees perceive their work environment as beneficial and positive (Mukherjee and Malhotra, 2006; Jong, 2016). These factors can motivate medical staff to devote themselves fully to their work and gain more self-affirmation, which ultimately improves overall job satisfaction.

In terms of job demands, we found that the job satisfaction decreased with increased work-family conflict, consistent with Tran and Yuan’s conclusion (Tran, 2022; Yuan et al., 2022). This may be attributable to the fact that medical staff who have a congenial family atmosphere are able to concentrate better on their work and experience less work pressure (Wu et al., 2021), thus obtaining more job satisfaction (Bruck et al., 2002). In addition to work, family is also an indispensable part of people’s life. Different from other groups, doctors and nurses work in a high-pressure work environment; therefore, hospital managers should help minimize the work-family conflict caused by work pressure as much as possible and pay attention to the work-life balance of employees.

Finally, this study found that job emotional demands also showed a negative impact on job satisfaction. Emotional demands are part of job demands (Brotheridge and Lee, 2002; Martínez-Iñigo et al., 2009), and satisfaction of employees’ emotional demands is also considered a necessary precondition for good job performance. Medical staff who they have a good relationship with patients and family members and whose emotional demands are met will perceive less psychological stress and greater job satisfaction. This may be because a harmonious doctor–patient relationship helps to make them more proactive and optimistic, have greater drive and passion for their work, which helps improve job satisfaction.

### Career Calling and Job Satisfaction

In this study, with high career calling predicted greater job satisfaction (H_3_). Previous studies have consistently demonstrated a strong positive correlation between calling and satisfaction across different occupations (Wrzesniewski et al., 1997; Hall and Chandler, 2005; Duffy et al., 2012a; Conway et al., 2015). This phenomenon may be because career calling among medical staff inculcates a sense of identity and belonging to their work. Individuals who feel a strong sense of career calling are more enthusiastic about their work, so they have a stronger sense of the meaning and value of work (Liang, 2020). Research has found that people with a sense of calling tend to have more precise goals and values, which enable them to be better engaged in their work (Bunderson and Thompson, 2009). Correspondingly, medical staff with a high level of career calling often have an apparent self-awareness of themselves and regard work as self-realization, which helps motivate individuals to pursue growth and development, to treat work positively, and achieve more job satisfaction. For a long time, medical personnel have been considered people under high pressure and high workload. However, serving people’s health has its attributes of sacrifice and dedication. Therefore, it is more important to cultivate a stronger sense of professional identity and mission, improve job satisfaction, and make them more proactive in serving people’s health. Research has shown that by enhancing the meaning of work (Bunderson and Thompson, 2009; Hirschi, 2012) and reaffirming the social status of their occupations (Cartwright and Holmes, 2006), it is possible to reduce employee cynicism and maintain their motivation to participate in work during uncertain and turbulent times. For instance, during the COVID-19 pandemic, countless medical workers, regardless of their safety, persisted in fighting the epidemic and were inspired by their inner career calling (Zhou, 2022). This demonstrates the significance and importance of career calling in the medical profession.

### The Dual Role of Career Calling

On one hand, we found that job resources and demands promoted job satisfaction through strengthening the sense of career calling (H_4a_ and H_4b_). In other words, more job resources and more moderate job demands predict a higher level of career calling, and therefore predict a higher level of job satisfaction, which was in line with the findings of other studies (Duffy and Sedlacek, 2007; Peng et al., 2019; Moll-Khosrawi et al., 2021). Moll-Khosrawi et al. (2021) studied the relationship between intrinsic motivation and job satisfaction using the self-determination theory. They argued that intrinsic motivation and autonomous regulation had effects on job satisfaction, which was positively correlated with intrinsic motivation and negatively correlated with amotivation, mirroring the conclusions of the present study. According to the self-determination theory, external factors at work can boost job satisfaction by increasing motivation level (Deci and Ryan, 2000). Environmental factors in an organization, job resources, and job demands influence the motives of employees, and hence guide their behaviors (Duffy et al., 2015). Thus, this theory can explain the effects of job resources and demands on the job satisfaction of health workers. As a kind of intrinsic motivation and autonomous regulation, “career calling” could affect one’s behaviors under the influence of circumstances. In other words, job resources and demands that meet the needs of health workers would lead to a rise in the sense of career calling, and hence improve job satisfaction. Besides, it is generally believed that people with a strong sense of career calling tend to experience more job meaning and satisfaction by focusing on the lofty goals and career development of their jobs, rather than financial benefits (Wrzesniewski et al., 1997; Bunderson and Thompson, 2009). Therefore, health professionals with a strong sense of career calling are more liable to pursue career development, and are therefore better at leveraging job resources, adapting to or moderating work pressure, creating more and better value, and gaining a higher level of job satisfaction (Duffy et al., 2011).

On the other hand, this study revealed that career calling had moderating effect in the paths of “job resources → career calling → job satisfaction” and “job demands → career calling → job satisfaction.” When the level of career calling is higher, the boosting effect of job resources on job satisfaction is more significant (H_5a_), and when the level of career calling is low, the inhibitory effect of job demands on job satisfaction is more significant (H_5b_), which was in line with the conclusions drawn by Peng et al. (2020). This result could be explained using the self-determination theory: when environmental elements such as job resources and job demands are not conducive to personal fulfillment of an individual, his/her behaviors are liable to be affected by these kinds of external motivations, and the functions of career calling, that is, intrinsic motivation and autonomous regulation, would be inhibited (Deci and Ryan, 2000). Health professionals who are deeply moderated by the sense of career calling pursue progress and appreciate organizational resources, which means that job resources provided by hospitals are more likely to be valued and properly utilized by them and that they can perceive a stronger sense of fulfillment and hence a stronger sense of job satisfaction (Hirschi, 2011). However, for those who perceive a weak sense of career calling, working in healthcare might simply be a means of earning a living. Thus, they might not value or fully utilize the job resources and are prone to be intolerant of job demands and be bored or even dissatisfied with the job. This further demonstrated that the job satisfaction of health workers is closely associated with career calling and provided empirical evidence of the effect of job resources of health workers on their job satisfaction.

This study found that career calling plays a dual role among job resources, job demands, and job satisfaction. Therefore, this study attempted to construct a “Model of Dual Effects of career calling.” Our findings indicated that career calling is a dual concept. The level of career calling is affected by factors such as the external environment and work pressure. It always adjusts and influences the relationship between the external environment and job satisfaction.

### Theoretical Implications

This study has several main contributions to the current knowledge. First, our research further explored the impact mechanism of medical staff’s job demands and job resources on job satisfaction. It deepened the intermediary mechanism between the two paths of “job resources-job satisfaction” and “job requirements-job satisfaction” for medical staff understanding. Second, this study is the practical application of the JD-R model in the medical and health field. Verifying the JD-R model increases our understanding of the JD-R model and expands the application boundary of the job requirement-resource model. The relationship between the job resource-demand model and satisfaction has received much attention. The research on its potential impact mechanism and process has been neglected to a certain extent (Ju and Shao, 2004; Xia and Lu, 2014). Third, our findings validate and enrich the notion that a factor can act as both a mediator and a moderator (MacKinnon, 2008; Dicke et al., 2014; Attar-Schwartz, 2015; Liu and Li, 2017; Peng et al., 2021). We constructed the “dual role model of occupational calling” for the first time and found that career calling played a dual role among job resources, job requirements and job satisfaction. Our study expands on existing research on the mechanism of action of career calling and responds positively to the call to investigate the antecedents of career calling in different contexts and occupations (Duffy et al., 2018; Lysova et al., 2018, 2019; Thompson and Bunderson, 2019). Fourth, this study also confirmed that job satisfaction is positively correlated with intrinsic motivation and negatively correlated with de-motivation, this is consistent with self-determination theory.

### Practical Implications

This study showed that job resources can affect the job satisfaction of medical staff, among which social support, performance feedback, and working conditions had more significant effect. Job demands, especially work-family conflict and job emotional demands had a significant negative impact on the job satisfaction of medical staff. These findings have important implications for improving the job satisfaction of medical staff and promoting the transformation and improvement of hospital management. On the one hand, hospital managers should focus on providing more material and moral support for medical staff to ensure the quality of medical services. On the other hand, colleagues should support, encourage, and help each other obtain more moral support and humanistic care. In addition, medical staff should also be provided with opportunities to learn new clinical technologies. Moreover, a fair and competitive salary system and a fair and reasonable promotion system should be established to meet the professional development expectations of medical staff. Our findings also suggest that the government and hospitals should invest more funds to improve the working conditions of various aspects of the hospital and provide a comfortable and secure working environment.

From the perspective of job demands, healthcare workers must reduce or even avoid work–family conflicts caused by work stress (Batool et al., 2017; Wang et al., 2017b; Habibian et al., 2018). Hospital managers can further improve and innovate the management system according to the working environment of the hospital. They must formulate scientific and reasonable work demands, reduce ineffective work content, moderately reduce the working hours of medical staff, reasonably mobilize work resources, allocate tasks, and ensure adequate rest time (Zhou et al., 2020). At the same time, it is also necessary to improve the communication ability of medical staff, build a harmonious doctor–patient relationship, and avoid excessive work demands and doctor–patient conflicts that reduce job satisfaction.

In addition, the mediating and moderating role of career calling should also be considered. Research showed that career calling as a work-related resource is U-shaped (Zhou et al., 2020), dynamic, and teachable (Dobrow, 2013; Duffy et al., 2014). Training programs can offer the possibility of improving career calling. Therefore, hospitals should implement career calling education in the process of calling training, attach importance to motivating employees’ inner beliefs and sense of mission, and strengthen the education of the value and significance of career. In addition, hospital managers can gradually introduce the assessment of the career calling of medical staff during recruitment and inspection using various forms such as questionnaires, psychological tests, and interviews, and draw corresponding conclusions. However, it is worth noting that our research results show that when the occupational vocation of medical staff is high, the effect of job resources in promoting job satisfaction is more significant; when the occupational vocation of medical staff is low, the inhibitory effect of job requirements is more pronounced. Therefore, managers should recognize the different levels of the professional calling of medical personnel.

### Limitations and Future Studies

This study has some potential limitations. First, this was a cross-sectional study, which means only correlations, rather than causal relationships, can be determined. Therefore, longitudinal studies like a crossover study or a research design involving two-time points are required to determine causal relationships. The second limitation is sample representativeness. This study was conducted in Hangzhou, which is a relatively developed city. Therefore, the status of job resources, demands, and calling might be different in less developed areas, and our results may not be entirely generalizable to other areas. Future studies should expand the geographical scope to include other provinces to make the results more representative. Third, this study focused on overall satisfaction without looking at more detailed outcome variables. Finally, the variables involved in the questionnaire survey of this study were all generated by the self-report of medical staff, and there is inevitably a particular homology bias (CMV), which may not reflect the experience and opinions of medical staff to some extent. In future research, specific dimensions in the job satisfaction scale could be introduced, such as satisfaction with the job itself, satisfaction with work conditions, satisfaction with interpersonal relationships, satisfaction with management, to provide more in-depth insights.

## Conclusion

This study demonstrated that the health professionals in Hangzhou had a moderate degree of overall job satisfaction and that job resources, demands, and calling all had effects on job satisfaction. Job resources and career calling contributed to positive effects on job satisfaction, which increased with an increase in scores for social support, performance feedback, and working conditions; job demands showed a negative effect on job satisfaction, which decreased with an increase in scores for work-family conflict and emotional requirements for work; career calling played the roles of moderator and partial mediator in the paths between job resources, demands, and job satisfaction. Therefore, healthcare administrators and hospital managers should monitor the status of job resources for health workers, provide timely and effective performance feedback, and provide adequate social support and working conditions. Moreover, they need to facilitate work-life balance, take cognizance of the emotional needs of health workers, and impose reasonable requirements. Additionally, to ease the strain and reduce conflicts, managers could try to improve job satisfaction by developing their sense of career calling.

## Data Availability Statement

The raw data supporting the conclusions of this article will be made available by the authors, without undue reservation.

## Ethics Statement

The studies involving human participants were reviewed and approved by Institutional Review Board of Hanghzou Normal University (approval number: 2021-1146). The patients/participants provided their written informed consent to participate in this study.

## Author Contributions

XH: conceptualization, methodology, and software. HC: visualization and investigation. YG: formal analysis and writing—original draft preparation. JW: software and validation. XW: supervision. TS: writing—review and editing. All authors contributed to the article and approved the submitted version.

## Funding

This work was supported by the National Natural Science Foundation of China Project (grant number: 71974050) and the Soft Science Research Program of Zhejiang Provincial Science and Technology Plan (grant number: 2021C35012).

## Conflict of Interest

The authors declare that the research was conducted in the absence of any commercial or financial relationships that could be construed as a potential conflict of interest.

## Publisher’s Note

All claims expressed in this article are solely those of the authors and do not necessarily represent those of their affiliated organizations, or those of the publisher, the editors and the reviewers. Any product that may be evaluated in this article, or claim that may be made by its manufacturer, is not guaranteed or endorsed by the publisher.

## Abbreviations

JD-R: Job Demands-Resources
SEM: Structural Equation Model
GFI: goodness of fit
AGFI: adjusted goodness of fit
NFI: normed fit index
CFI: comparative fit index
TLI: Tucker–Lewis index
RMSEA: root mean square error of approximation

## References

[ref1] Attar-Schwartz S. (2015). Emotional closeness to parents and grandparents: a moderated mediation model predicting adolescent adjustment. Am. J. Orthopsychiatry 85, 495–503. doi: 10.1037/ort0000082, PMID: 26237053

[ref2] Bagheri Hossein Abadi M. Taban E. Khanjani N. Naghavi Konjin Z. Khajehnasiri F. Samaei S. E. (2020). Relationships between job satisfaction and job demand, job control, social support, and depression in Iranian nurses. J. Nurs. Res. 29:e143. doi: 10.1097/jnr.0000000000000410, PMID: 33156140

[ref3] Bakker A. B. Demerouti E. (2007). The job demands-resources model: state of the art. J. Manag. Psychol. 22, 309–328. doi: 10.1108/02683940710733115, PMID: 31861812

[ref4] Bakker A. B. Demerouti E. Verbeke W. (2004). Using the job demands-resources model to predict burnout and performance. Hum. Resour. Manag. 43, 83–104. doi: 10.1002/hrm.20004

[ref5] Batool I. Hussain S. Bajwa R. S. (2017). Job satisfaction and marital adjustment among paramedical: mediating role of work family conflict. J. Bus. Soc. Rev. Emerg. Econ. 3, 61–74. doi: 10.26710/jbsee.v3i1.37

[ref6] Bollen K. A. Stine R. A. (1992). Bootstrapping goodness-of-fit measures in structural equation models. Sociol. Methods Res. 21, 205–229. doi: 10.1177/0049124192021002004

[ref7] Brotheridge C. M. Lee R. T. (2002). Testing a conservation of resources model of the dynamics of emotional labor. J. Occup. Health Psychol. 7, 57–67. doi: 10.1037/1076-8998.7.1.57, PMID: 11827234

[ref8] Bruck C. S. Allen T. D. Spector P. E. (2002). The relation between work–family conflict and job satisfaction: a finer-grained analysis. J. Vocat. Behav. 60, 336–353. doi: 10.1006/jvbe.2001.1836

[ref9] Bunderson J. S. Thompson J. A. (2009). The call of the wild: zookeepers, callings, and the double-edged sword of deeply meaningful work. Adm. Sci. Q. 54, 32–57. doi: 10.2189/asqu.2009.54.1.32

[ref10] Cartwright S. Holmes N. (2006). The meaning of work: The challenge of regaining employee engagement and reducing cynicism. Hum. Resour. Manag. Rev. 16, 199–208. doi: 10.1016/j.hrmr.2006.03.012

[ref11] Chang P.-C. Rui H. Wu T. (2021). Job autonomy and career commitment: a moderated mediation model of job crafting and sense of calling. SAGE Open 11:215824402110041. doi: 10.1177/21582440211004167

[ref12] Chen X. Y. You L. L. Wang H. Q. Lian J. Yang L. Liu M. C.. (2021). Job satisfaction and influencing factors among essential public health practitioners in primary care [Chinese]. Chin. Gen. Pract. 24, 3597–3601. doi: 10.12114/j.issn.1007-9572.2021.00.285

[ref13] Conway N. Clinton M. Sturges J. Budjanovcanin A. (2015). Using self-determination theory to understand the relationship between calling enactment and daily well-being. J. Organ. Behav. 36, 1114–1131. doi: 10.1002/job.2014

[ref14] Crawford E. R. Lepine J. A. Rich B. L. (2010). Linking job demands and resources to employee engagement and burnout: a theoretical extension and meta-analytic test. J. Appl. Psychol. 95, 834–848. doi: 10.1037/a0019364, PMID: 20836586

[ref15] Deci E. L. Ryan R. M. (2000). The “what” and “why” of goal pursuits: human needs and the self-determination of behavior. Psychol. Inq. 11, 227–268. doi: 10.1207/S15327965PLI1104_01, PMID: 20204932

[ref16] Deci E. L. Ryan R. M. (2012). Self-determination theory.

[ref17] Demerouti E. Bakker A. B. Nachreiner F. Schaufeli W. B. (2001). The job demands-resources model of burnout. J. Appl. Psychol. 86, 499–512. doi: 10.1037/0021-9010.86.3.499, PMID: 11419809

[ref18] Dicke T. Parker P. D. Marsh H. W. Kunter M. Schmeck A. Leutner D. (2014). Self-efficacy in classroom manage-ment, classroom disturbances, and emotional exhaustion: a moderated mediation analysis of teacher candidates. J. Educ. Psychol. 106, 569–583. doi: 10.1037/a0035504

[ref19] Dobrow S. R. (2013). Dynamics of calling: a longitudinal study of musicians. J. Organ. Behav. 34, 431–452. doi: 10.1002/job.1808

[ref20] Dobrow S. R. Tosti-Kharas J. (2011). Calling: the development of a scale measure. Pers. Psychol. 64, 1001–1049. doi: 10.1111/j.1744-6570.2011.01234.x, PMID: 34435850

[ref21] Drüge M. Schladitz S. Wirtz M. A. Schleider K. (2021). Psychosocial burden and strains of pedagogues-using the job demands-resources theory to predict burnout, job satisfaction, general state of health, and life satisfaction. Int. J. Environ. Res. Public Health 18:7921. doi: 10.3390/ijerph18157921, PMID: 34360214 PMC8345630

[ref22] Duffy R. D. Allan B. A. Bott E. M. (2012a). Calling and life satisfaction among undergraduate students: investigating mediators and moderators. J. Happiness Stud. 13, 469–479. doi: 10.1007/s10902-011-9274-6

[ref23] Duffy R. D. Allan B. A. Bott E. M. Dik B. J. (2014). Does the source of a calling matter? External summons, destiny, and perfect fit. J. Career Assess. 22, 562–574. doi: 10.1177/1069072713514812

[ref24] Duffy R. D. Autin K. L. Allan B. A. Douglass R. P. (2015). Assessing work as a calling: an evaluation of instruments and practice recommendations. J. Career Assess. 23, 351–366. doi: 10.1177/1069072714547163

[ref25] Duffy R. D. Bott E. M. Allan B. A. Torrey C. L. Dik B. J. (2012b). Perceiving a calling, living a calling, and job satisfaction: testing a moderated, multiple mediator model. J. Couns. Psychol. 59, 50–59. doi: 10.1037/a0026129, PMID: 22059426

[ref26] Duffy R. D. Dik B. J. Douglass R. P. England J. W. Velez B. L. (2018). Work as a calling: a theoretical model. J. Couns. Psychol. 65, 423–439. doi: 10.1037/cou0000276, PMID: 29999369

[ref27] Duffy R. D. Dik B. J. Steger M. F. (2011). Calling and work-related outcomes: career commitment as a mediator. J. Vocat. Behav. 78, 210–218. doi: 10.1016/j.jvb.2010.09.013

[ref28] Duffy R. D. Sedlacek W. E. (2007). The presence of and search for a calling: connections to career development. J. Vocat. Behav. 70, 590–601. doi: 10.1016/j.jvb.2007.03.007

[ref29] Duffy R. D. Sedlacek W. E. (2010). The salience of a career calling among college students: exploring group differences and links to religiousness, life meaning, and life satisfaction. Career Dev. Q. 59, 27–41. doi: 10.1002/j.2161-0045.2010.tb00128.x

[ref30] Enders C. K. (2005). An SAS macro for implementing the modified Bollen-Stine bootstrap for missing data: implementing the bootstrap using existing structural equation modeling software. Struct. Equ. Model. 12, 620–641. doi: 10.1207/s15328007sem1204_6

[ref31] Flores N. Moret-Tatay C. Gutiérrez-Bermejo B. Vázquez A. Jenaro C. (2021). Assessment of occupational health and job satisfaction in workers with intellectual disability: a job demands-resources perspective. Int. J. Environ. Res. Public Health 18:2072. doi: 10.3390/ijerph18042072, PMID: 33672616 PMC7924175

[ref32] Fors Brandebo M. Sterberg J. Berglund A. K. (2019). The impact of constructive and destructive leadership on Soldier's job satisfaction. Psychol. Rep. 122, 1068–1086. doi: 10.1177/0033294118771542, PMID: 29699471

[ref33] Ghanayem M. Srulovici E. Zlotnick C. (2020). Occupational strain and job satisfaction: the job demand-resource moderation-mediation model in haemodialysis units. J. Nurs. Manag. 28, 664–672. doi: 10.1111/jonm.12973, PMID: 32034951

[ref34] Gomez-Mejia L. R. (1990). Increasing productivity: performance appraisal and reward systems. Pers. Rev. 19, 21–26. doi: 10.1108/00483489010138759, PMID: 10145492

[ref35] Grant A. M. Berry J. W. (2011). The necessity of others is the mother of invention: intrinsic and prosocial motivations, perspective taking, and creativity. Acad. Manag. J. 54, 73–96. doi: 10.5465/amj.2011.59215085

[ref36] Grant A. M. Dutton J. E. Rosso B. D. (2008). Giving commitment: employee support programs and the prosocial sensemaking process. Acad. Manag. J. 51, 898–918. doi: 10.5465/amj.2008.34789652

[ref37] Greene A. M. Robbins M. (2015). The cost of a calling? Clergywomen and work in the church of England. Gend. Work. Organ. 22, 405–420. doi: 10.1111/gwao.12101

[ref38] Habibian H. Babakhanian M. Mohammadi G. Deljo F. Moradabad Z. S. Darvishbaghal N. R.. (2018). Relationship Between work-family conflict and job satisfaction of medical staff after implementing the health system development plan. Crisis 5:12. doi: 10.5812/mejrh.57141

[ref39] Hall D. T. Chandler D. E. (2005). Psychological success: when the career is a calling. J. Organ. Behav. 26, 155–176. doi: 10.1002/job.301

[ref40] Hirschfeld R. R. (2000). Does revising the intrinsic and extrinsic subscales of the Minnesota satisfaction questionnaire short form make a difference? Educ. Psychol. Meas. 60, 255–270. doi: 10.1177/00131640021970493

[ref41] Hirschi A. (2011). Callings in career: a typological approach to essential and optional components. J. Vocat. Behav. 79, 60–73. doi: 10.1016/j.jvb.2010.11.002

[ref42] Hirschi A. (2012). Callings and work engagement: moderated mediation model of work meaningfulness, occupational identity, and occupational self-efficacy. J. Couns. Psychol. 59, 479–485. doi: 10.1037/a0028949, PMID: 22774870

[ref43] Hu Y. Y. Liu Y. X. Li N. Yang Y. M. Wang W. J. (2021). The influence of elementary and secondary school teachers' character strengths on work engagement: the mediating role of professional calling [Chinese]. Contemp. Educ. Sci. 9, 80–87.

[ref44] Jiang H. T. Zhang F. Liang J. Y. (2019). Relationship between job satisfaction and job burnout as well as turnover intention among oncology nurses [Chinese]. Indust. Health Occup. Dis. 45, 268–270. 274. doi: 10.13692/j.cnki.gywsyzyb.2019.04.008

[ref45] Johnson J. V. Hall E. M. (1988). Job strain, work place social support, and cardiovascular disease: a cross-sectional study of a random sample of the Swedish working population. Am. J. Public Health 78, 1336–1342. doi: 10.2105/AJPH.78.10.1336, PMID: 3421392 PMC1349434

[ref46] Jong J. (2016). The role of performance feedback and job autonomy in mitigating the negative effect of role ambiguity on employee satisfaction. Public Perform. Manag. Rev. 39, 814–834. doi: 10.1080/15309576.2015.1137771

[ref47] Ju X. Shao L. C. (2004). Job requirements for burnout-resource model [Chinese]. Appl. Psychol. 10, 58–62.

[ref48] Karasek R. A. Jr. (1979). Job demands, job decision latitude, and mental strain: implications for job redesign. Adm. Sci. Q. 24, 285–308. doi: 10.2307/2392498, PMID: 24274148

[ref49] Li M. (2006). Research on Teacher's Job Burnout Based on JDR Theory [Chinese]. Beijing, China: Capital Normal University.

[ref50] Li J. N. (2011). *The Study on Job Demands-Resources Model of Work Engagement among Nurses in Henan Ruzhou [Chinese]*. Master degree, Zhengzhou University.

[ref51] Li D. B. (2021). The influence of career identity on rural teachers' tendency to leave the profession - appreciating the chain mediating role of social support and calling [Chinese]. Educ. Res. Month. 9, 28–36. doi: 10.16477/j.cnki.issn1674-2311.2021.09.004

[ref52] Liang W. Y. (2020). Job demands, job resources and teachers job satisfaction: an empirical study based on the Shanghai data from the TALIS 2018 results [Chinese]. Educ. Res. 41, 102–115.

[ref53] Liu K. (2019). *Research on the Relationship between Employees’ Professional Resources, Sense of Professional Mission and Subjective Professional Success* [Chinese]. Master, Nanjing Normal University.

[ref54] Liu D. N. Li D. P. (2017). Parenting styles and adolescent internet addiction: an examination of the mediating and moderating roles of ego-resiliency [Chinese]. J. Psychol. Sci. 40, 1385–1391. doi: 10.16719/j.cnki.1671-6981.20170617

[ref55] Lopez-Martin E. Topa G. (2019). Organizational culture and job demands and resources: their impact on employees' wellbeing in a multivariate multilevel model. Int. J. Environ. Res. Public Health 16:3006. doi: 10.3390/ijerph16173006, PMID: 31438459 PMC6747151

[ref56] Lysova E. I. Dik B. J. Duffy R. D. Khapova S. N. Arthur M. B. (2019).Calling and Careers: New Insights and Future Directions. J. Voc. Behav. Volu. 11, 1–6.

[ref57] Lysova E. I. Jansen P. G. Khapova S. N. Plomp J. Tims M. (2018). Examining calling as a double-edged sword for employability. J. Vocat. Behav. 104, 261–272. doi: 10.1016/j.jvb.2017.11.006

[ref58] Mache S. Vitzthum K. (2012). Are health professionals' working conditions and job satisfaction associated to patient satisfaction? Dtsch Med Wochenschr 137:A207. doi: 10.1055/s-0032-1323370

[ref59] MacKinnon D. P. (2008). Introduction to Statistical Mediation Analysis. New York, NY: Erlbaum.

[ref60] Mao Y. H. Xu S. S. Huang L. S. (2019). Pediatric nurses’ calling and influencing factors in China. Chin. Nurs. Manag. 19, 1343–1346. doi: 10.3969/j.issn.1672-1756.2019.09.013

[ref61] Martínez-Iñigo D. Totterdell P. Alcover C. M. Holman D. (2009). The source of display rules and their effects on primary health care professionals’ well-being. Span. J. Psychol. 12, 618–631. doi: 10.1017/S1138741600001980, PMID: 19899662

[ref62] Moll-Khosrawi P. Zimmermann S. Zoellner C. Schulte-Uentrop L. (2021). Understanding why all types of motivation are necessary in advanced anaesthesiology training levels and how they influence job satisfaction: translation of the self-determination theory to healthcare. Healthcare 9:262. doi: 10.3390/healthcare9030262, PMID: 33804576 PMC7999734

[ref63] Mukherjee A. Malhotra N. (2006). Does role clarity explain employee-perceived service quality? A study of antecedents and consequences in call centres. Int. J. Serv. Ind. Manag. 17, 444–473. doi: 10.1108/09564230610689777

[ref64] Peng S. Niu G. F. Wang X. Zhang H. P. Hu X. E. (2021). Parental autonomy support and adolescents’ positive emotional adjustment: mediating and moderating roles of basic need satisfaction. Psychol. Dev. Educ. 37, 240–248. doi: 10.16187/j.cnki.issn1001-4918.2021.02.11

[ref65] Peng J. Zhang J. Zhang Y. Gong P. (2019). Relative deprivation and job satisfaction in Army officers: a moderated mediation model. Work 62, 49–58. doi: 10.3233/WOR-182841, PMID: 30741713

[ref66] Peng J. Zhang J. Zheng L. Guo H. Miao D. Fang P. (2020). Career calling and job satisfaction in army officers: a multiple mediating model analysis. Psychol. Rep. 123, 2459–2478. doi: 10.1177/0033294119862990, PMID: 31307282

[ref67] Ping R. A. (1995). A parsimonious estimating technique for interaction and quadratic latent variables. J. Mark. Res. 32, 336–347. doi: 10.1177/002224379503200308

[ref68] Potocka A. Waszkowska M. (2013). Application of job demands-resources model in research on relationships between job satisfaction, job resources, individual resources and job demands [Polish]. Med. Pr. 64, 217–225. doi: 10.13075/mp.5893/2013/0018 23829066

[ref69] Scanlan J. N. Still M. (2019). Relationships between burnout, turnover intention, job satisfaction, job demands and job resources for mental health personnel in an Australian mental health service. BMC Health Serv. Res. 19:62. doi: 10.1186/s12913-018-3841-z, PMID: 30674314 PMC6343271

[ref70] Scarpello V. Campbell J. (1983). Job satisfaction: are All the parts there? Pers. Psychol. 36, 577–600. doi: 10.1111/j.1744-6570.1983.tb02236.x, PMID: 35418211

[ref71] Shi J. M. Zhao S. S. Wu Y. H. (2018). Spiritual leadership and career calling: a research based on self-determination theory economic management. Bus. Manag. J. 40, 138–152. doi: 10.19616/j.cnki.bmj.2018.12.009

[ref72] Singh J. (1998). Striking a balance in boundary-spanning positions: an investigation of some unconventional influences of role stressors and job characteristics on job outcomes of salespeople. J. Mark. 62, 69–86. doi: 10.1177/002224299806200305

[ref73] Su X. H. Bozeman B. (2009). Do expectations meet aspirations? The relation of public managers’ job choice motives to job satisfaction. Int. Rev. Public Adm. 14, 1–9. doi: 10.1080/12294659.2009.10805143

[ref74] Thompson J. A. Bunderson J. S. (2019). Research on work as a calling… and how to make it matter. Annu. Rev. Organ. Psych. Organ. Behav. 6, 421–443. doi: 10.1146/annurev-orgpsych-012218-015140, PMID: 27896967

[ref75] Tims M. Bakker A. B. Derks D. (2012). Development and validation of the job crafting scale. J. Vocat. Behav. 80, 173–186. doi: 10.1016/j.jvb.2011.05.009, PMID: 35330727

[ref76] Tran Q. H. N. (2022). “Exploring Relationships Among Overload Stress, Work-Family Conflict, Job Satisfaction, Person–Organisation Fit and Organisational Commitment in Public Organizations” in Public Organization Review, 1–17.

[ref77] Wang H. Qian Y. Wang X. H. (2017a). Development and validation studies of medical staff job satisfaction assessment scale [Chinese]. Chin. Hosp. Manag. 37, 14–17.

[ref78] Wang Y. Wei Z. F. Wang L. (2012). An empirical study on the impact of job resources and job engagement on knowledge sharing [Chinese]. Sci. Technol. Manag. Res. 32, 150–153. doi: 10.3969/j.issn.1000-7695.2012.24.034

[ref79] Wang S. Xie F. Z. Shi Y. Meng D. X. Zhang S. E. Wang J. H.. (2017b). Development of a clinician's job demand-resource scale and its impact on work-family conflict [Chinese]. Chin. Hosp. Manag. 37, 63–65.

[ref80] Wanous J. P. Reichers A. E. Hudy M. J. (1997). Overall job satisfaction: how good are single-item measures? J. Appl. Psychol. 82, 247–252. doi: 10.1037/0021-9010.82.2.247, PMID: 9109282

[ref81] Wrzesniewski A. Mccauley C. Rozin P. Schwartz B. (1997). Jobs, careers, and callings: People's relations to their work. J. Res. Pers. 31, 21–33. doi: 10.1006/jrpe.1997.2162

[ref82] Wu L. Zhou S. Gao Y. Wang X. (2021). A study on job satisfaction, stressors and job burnout of nurses in a third-class hospital in Haikou. Open J. Nurs. 11, 600–609. doi: 10.4236/ojn.2021.117051

[ref83] Xia F. B. Lu X. D. (2014). Job requirements-new progress in resource theory research [Chinese]. Spec. Zone Econ., 77–78.

[ref84] Xian M. T. (2019). Work resources-stress appraisal model of male nurses’ burnout [Chinese]. master degree (master thesis). Southern Medical University.

[ref85] Xian M. T. Zhai H. M. (2019). Role of work resources in relationship between job demands and job burnout of male nurses [Chinese]. Occup. Health 35, 2759–2763. doi: 10.13329/j.cnki.Zyyjk.2019.0782

[ref86] Xie C. L. (2017). The Effect of Job Requirements on Job Burnout: An Empirical Study of the Moderating Effect of Job Resources and Career Callings [Chinese]. Kunming, Yunnan, China: Yunnan University of Finance and Economics.

[ref87] Xu L. Gao L. Chen Y. Zhai R. H. (2020). Relationship between perceived stress and psychological resilience of first-line nurses in hospitals designated for treatment of patients with COVID-19: the mediating role of professional Mission [Chinese]. Chin. Med. Ethics 33, 487–491. doi: 10.12026/j.issn.1001-8565.2020.04.22

[ref88] Ye Z. P. Feng X. (2009). Significance of evaluation and measurement of medical Personnel's job satisfaction [Chinese]. Northwest Med. Edu. 4, 704–707. doi: 10.13555/j.cnki.c-me.2009.04.074

[ref89] Yeh H. J. (2015). Job demands, job resources, and job satisfaction in East Asia. Soc. Indic. Res. 121, 47–60. doi: 10.1007/s11205-014-0631-9, PMID: 35031046

[ref90] Yuan Q. (2018). Relationship between the JD-R models and teachers’ job burnout: the five personality mechanisms [Chinese]. master degree (master thesis). Hunan Normal University.

[ref91] Yuan T. Liang L. Ren H. Hu Y. Qin Z. Fei J.. (2022). Age moderates the effect of work-family conflict on life satisfaction among Chinese female employees: A propensity score matching method. Personal. Individ. Differ. 185:111279. doi: 10.1016/j.paid.2021.111279

[ref92] Zhang T. Feng J. Jiang H. Shen X. Pu B. Gan Y. (2021). Association of professional identity, job satisfaction and burnout with turnover intention among general practitioners in China: evidence from a national survey. BMC Health Serv. Res. 21:382. doi: 10.1186/s12913-021-06322-6, PMID: 33902579 PMC8074426

[ref93] Zhang L. G. Li L. Sun Y. L. (2020). A study of the relationships between occupational stress, career calling and occupational burnout among primary teachers [Chinese]. Zhonghua Lao Dong Wei Sheng Zhi Ye Bing Za Zhi 38, 107–110. doi: 10.3760/cma.j.issn.1001-9391.2020.02.006, PMID: 32306672

[ref94] Zhou J. (2022). How does COVID-19 pandemic strength influence work fatigue? The mediating role of occupational calling. Curr. Psychol. 1–13. doi: 10.1007/s12144-022-02846-0 PMC9478163 36128516

[ref95] Zhou J. Zhang J. W. Xuan X. Y. (2020). The curvilinear relationship between career calling and work fatigue: a moderated mediating model. Front. Psychol. 11:583604. doi: 10.3389/fpsyg.2020.583604, PMID: 33192910 PMC7661552

