# Supplementary material for: Career Calling as the Mediator and Moderator of Job Demands and Job Resources for Job Satisfaction in Health Workers: A Cross-Sectional Study
Source: Front Psychol. 2022 May 10;13:856997. doi: 10.3389/fpsyg.2022.856997 (PMC9127994; doi:10.3389/fpsyg.2022.856997)
Supplement: Supplementary file 1 [file Data_Sheet_1.docx]

# Appendix

Reliability of the survey

**Table 1. Split-half reliability of the scale**

| Sections of items | Number of items | Cronbach’s alpha | Split-half reliability coefficient |
| --- | --- | --- | --- |
| Job resources |  |  |  |
| Odd-numbered items | 10 | 0.927 |  |
| Even-numbered items | 9 | 0.929 |  |
| Total items | 19 |  | 0.909 |
| Job demands |  |  |  |
| Odd-numbered items | 6 | 0.796 |  |
| Even-numbered items | 5 | 0.745 |  |
| Total items | 11 |  | 0.764 |
| Calling |  |  |  |
| Odd-numbered items | 2 | 0.821 |  |
| Even-numbered items | 2 | 0.824 |  |
| Total items | 4 |  | 0.789 |

**Table 2. Cronbach’s alpha for the scale**

| Dimensions | Number of items | Cronbach’s alpha |
| --- | --- | --- |
| Job resources |  |  |
| Work autonomy | 3 | 0.913 |
| Social support | 5 | 0.915 |
| Performance feedback | 3 | 0.945 |
| Career development opportunities | 4 | 0.910 |
| Working conditions | 4 | 0.883 |
| Job resources in general | 19 | 0.964 |
| Job demands |  |  |
| Work operation requirements | 5 | 0.876 |
| Work-family conflict | 3 | 0.930 |
| Emotional requirements for work | 3 | 0.847 |
| Job demands in general | 11 | 0.888 |
| Career Calling |  |  |
| Career Calling | 4 | 0.913 |

Validity of the survey

5.1.2.2 Validity of the scale for job resources of health workers

(1) Content validity

**Table 3. Correlation between the score on each item and job resources**

| Content of items | Coefficient for correlation between the score on each item and the total score on job resources |
| --- | --- |
| B1 I determine the progress of work on my own | 0.695** |
| B2 I am entitled to decide how to carry out my work | 0.727** |
| B3 I can organize work-related affairs by myself | 0.729** |
| B4 I can seek help from leaders when encountering difficulties at work | 0.802** |
| B5 My leaders care about my physical and mental health | 0.843** |
| B6 My leaders are able to improve my relationships with colleagues | 0.855** |
| B7 I can get help from colleagues | 0.726** |
| B8 My colleagues are friendly to me | 0.699** |
| B9 I know precisely comments of leaders on my performance | 0.842** |
| B10 My leaders often offer feedbacks beneficial to career development | 0.871** |
| B11 Performance feedbacks from my leaders are well explained | 0.875** |
| B12 My job provides me with opportunities to learn professional skills including new clinical techniques and new projects | 0.826** |
| B13 The hospital I work at organizes various activities including lectures and training | 0.769** |
| B14 The hospital I work at organizes internal recruitment or selection for certain posts and titles | 0.787** |
| B15 The hospital I work at encourages us to apply for scientific projects | 0.733** |
| B16 In the department I work at, the technologies and equipment can meet the needs of patients | 0.765** |
| B17 The department I work at is staffed reasonably | 0.745** |
| B18 The department I work at has a good cultural atmosphere | 0.810** |
| B19 The hospital I work at can deal with violence happening in the workplace timely | 0.775** |

Note: ***P*<0.01

(2) Structural validity

**Table 4. Variance contribution rate of factors in the scale of job resources**

| Factor |  | Characteristic value | Variance contribution rate (%) | Cumulative variance contribution rate (%) |
| --- | --- | --- | --- | --- |
| 1 |  | 4.071 | 21.429 | 21.429 |
| 2 |  | 3.580 | 18.843 | 40.271 |
| 3 |  | 3.060 | 16.103 | 56.374 |
| 4 |  | 2.829 | 14.890 | 71.264 |
| 5 |  | 2.129 | 11.205 | 82.469 |

**Table 5. Rotated factor loading matrix for the scale of job resources**

| Content of items | Work autonomy | | Social support | Performance feedback | Career development opportunities | Working conditions |
| --- | --- | --- | --- | --- | --- | --- |
| B1 I determine the progress of work on my own | 0.840 | |  |  |  |  |
| B2 I am entitled to decide how to carry out my work | 0.891 | |  |  |  |  |
| B3 I can organize work-related affairs by myself | | 0.863 |  |  |  |  |
| B4 I can seek help from leaders when encountering difficulties at work | |  | 0.854 |  |  |  |
| B5 My leaders care about my physical and mental health | |  | 0.891 |  |  |  |
| B6 My leaders are able to improve my relationships with colleagues | |  | 0.914 |  |  |  |
| B7 I can get help from colleagues | |  | 0.867 |  |  |  |
| B8 My colleagues are friendly to me | |  | 0.825 |  |  |  |
| B9 I know precisely comments of leaders on my performance | |  |  | 0.766 |  |  |
| B10 My leaders often offer feedbacks beneficial to career development | |  |  | 0.757 |  |  |
| B11 Performance feedbacks from my leaders are well explained | |  |  | 0.711 |  |  |
| B12 My job provides me with opportunities to learn professional skills including new clinical techniques and new projects | |  |  |  | 0.545 |  |
| B13 The hospital I work at organizes various activities including lectures and training | |  |  |  | 0.846 |  |
| B14 The hospital I work at organizes internal recruitment or selection for certain posts and titles | |  |  |  | 0.833 |  |
| B15 The hospital I work at encourages us to apply for scientific projects | |  |  |  | 0.782 |  |
| B16 In the department I work at, the technologies and equipment can meet the needs of patients | |  |  |  |  | 0.591 |
| B17 The department I work at is staffed reasonably | |  |  |  |  | 0.789 |
| B18 The department I work at has a good cultural atmosphere | |  |  |  |  | 0.701 |
| B19 The hospital I work at can deal with violence happening in the workplace timely | |  |  |  |  | 0.699 |

5.1.2.2 Validity of the scale of job demands

(1) Content validity

**Table 6. Correlation between the score on each item and the total score on job demands**

| Content of items | Coefficient for correlation between the score on each item and the total score on job demands |
| --- | --- |
| C1 My job requires me to work in a fast-paced environment | 0.623** |
| C2 My job requires me to be very hard-working | 0.612** |
| C3 I have a heavy workload | 0.735** |
| C4 My job has strict requirements for the quality of work | 0.637** |
| C5 My job requires me to pay attention to the safety in operation | 0.472** |
| C6 Requirements of my job affected my family life | 0.732** |
| C7 Time spent in my job made me fail to undertake family responsibilities | 0.756** |
| C8 I failed to do what I wanted to do at home due to requirements of my job | 0.739** |
| C9 My job requires me to deal with various demands of patients or their relatives | 0.740** |
| C10 My work involves getting along with patients with poor compliance | 0.721** |
| C11 My work put me under heavy pressure | 0.777** |

Note: ***P*<0.01

(2) Structural validity

Results of the KMO Test and Bartlett’s Test of Sphericity indicated that factor analysis could be conducted, since the KMO was 0.859, and the test statistic for Bartlett’s Test of Sphericity was 8649.882 (*P*<0.01).

**Table 7. Variance contribution rate of factors in the scale of job demands**

| Factor | Characteristic value | Variance contribution rate (%) | Cumulative variance contribution rate (%) |
| --- | --- | --- | --- |
| 1 | 3.360 | 30.542 | 30.542 |
| 2 | 3.013 | 27.394 | 57.936 |
| 3 | 2.163 | 19.668 | 77.604 |

**Table 8. Rotated factor loading matrix for the scale of job demands**

| Content of items | Work operation requirements | Work-family conflict | Emotional requirements for work |
| --- | --- | --- | --- |
| C1 My job requires me to work in a fast-paced environment | 0.768 |  |  |
| C2 My job requires me to be very hard-working | 0.865 |  |  |
| C3 I have a heavy workload | 0.720 |  |  |
| C4 My job has strict requirements for the quality of work | 0.852 |  |  |
| C5 My job requires me to pay attention to the safety in operation | 0.732 |  |  |
| C6 Requirements of my job affected my family life |  | 0.891 |  |
| C7 Time spent in my job made me fail to undertake family responsibilities |  | 0.920 |  |
| C8 I failed to do what I wanted to do at home due to requirements of my job |  | 0.903 |  |
| C9 My job requires me to deal with various demands of patients or their relatives |  |  | 0.862 |
| C10 My work involves getting along with patients with poor compliance |  |  | 0.870 |
| C11 My work put me under heavy pressure |  |  | 0.607 |

5.1.2.3 Validity of the scale of calling

(1) Content validity

**Table 9. Correlation between the score on each item and calling**

| Content of items | Coefficient for correlation between the score on each item and calling |
| --- | --- |
| E1 I perceive a calling as a health worker | 0.821** |
| E2 I have found the career in which I have a calling | 0.839** |
| E3 I am trying to fathom the mission of health workers | 0.874** |
| E4 I am seeking my mission as a health worker | 0.876** |

Note: ***P*<0.01

(2) Structural validity

**Table 10. Rotated factor loading matrix for the scale of calling**

| Content of items | Calling |
| --- | --- |
| E1 I perceive a calling as a health worker | 0.861 |
| E2 I have found the career in which I have a calling | 0.859 |
| E3 I am trying to fathom the mission of health workers | 0.854 |
| E4 I am seeking my mission as a health worker | 0.839 |
